# A Novel Property of DNA – As a Bioflotation Reagent in Mineral Processing

**DOI:** 10.1371/journal.pone.0039316

**Published:** 2012-07-02

**Authors:** Balasubramanian Vasanthakumar, Honnavar Ravishankar, Sankaran Subramanian

**Affiliations:** Department of Materials Engineering, Indian Institute of Science, Bangalore, India; University of Quebect at Trois-Rivieres, Canada

## Abstract

Environmental concerns regarding the use of certain chemicals in the froth flotation of minerals have led investigators to explore biological entities as potential substitutes for the reagents in vogue. Despite the fact that several microorganisms have been used for the separation of a variety of mineral systems, a detailed characterization of the biochemical molecules involved therein has not been reported so far. In this investigation, the selective flotation of sphalerite from a sphalerite-galena mineral mixture has been achieved using the cellular components of *Bacillus* species. The key constituent primarily responsible for the flotation of sphalerite has been identified as DNA, which functions as a bio-collector. Furthermore, using reconstitution studies, the obligatory need for the presence of non-DNA components as bio-depressants for galena has been demonstrated. A probable model involving these entities in the selective flotation of sphalerite from the mineral mixture has been discussed.

## Introduction

The growing demand for mineral commodities across the world has led to the increased exploitation of lean grade ores with complex mineralogy, particularly for producing base metals. Additionally, depletion of the high grade mineral resources has resulted in the search for more advanced solutions to the problem of beneficiation of some refractory ores, in cases where conventional techniques are not efficient. These factors in combination with the more rigorous specifications for production of concentrates, stricter environmental legislation and a necessity to achieve lower operating costs has made it imperative to develop more effective flotation reagents.

Application of biotechnology in mineral processing has opened up immense possibilities for producing cleaner concentrates having acceptable grades and recoveries. Advances in molecular biology and genetic engineering have given an impetus for the characterization of biological entities of relevance to mineral processing. The bioflotation process may be defined as one in which microorganisms act as reagents, collectors or modifiers, to facilitate the selective separation of minerals [1]. The use of bioreagents as collectors invokes several interfacial aspects of the interacting biological and geological materials, viz., the physicochemical properties of the mineral surface, such as the atomic and electronic structure, the net charge/potential, the acid–base properties, and the wettability of the surface [2]. For the past two decades, several studies have been carried out on the use of microorganisms and their secretions viz., proteins and polysaccharides as environment friendly flotation reagents. The microorganism cell surface principally consists of functional groups derived from phospholipids, proteins and polysaccharides. Some of these induce hydrophobic properties, since they can adhere selectively to the mineral surface [3]. The emergence of mineral bioprocessing is reflected by the conferences exclusively dedicated to this topic [4], [5], [6], [7]. The bioflotation and bioflocculation processes of relevance to mineral beneficiation have been critically reviewed [1], [8]. These studies have led to the understanding that the interaction between specific microbial cells and mineral particles brings about significant changes in the chemistry of mineral surfaces, as well as the bacterial surfaces.

Despite the wealth of information that has been gathered so far, a detailed understanding of the nature and characteristics of the bioreagent responsible for the selective flotation of any given mineral from the mineral mixture has remained elusive. Such identification would constitute the first step in the large-scale generation and commercial exploitation of bioreagents involved in the flotation of minerals. In this communication, we report for the first time the identification and characterization of a bioreagent from *B.circulans* which plays a significant role in the selective flotation of sphalerite from a sphalerite-galena mixture. In addition, a plausible model for the interaction of this bioreagent with sphalerite leading to its selective flotation from the mineral mixture has been proposed.

## Results

### Buffering of *B.circulans* Cells Aids the Flotation of Sphalerite

**Figure 1:**
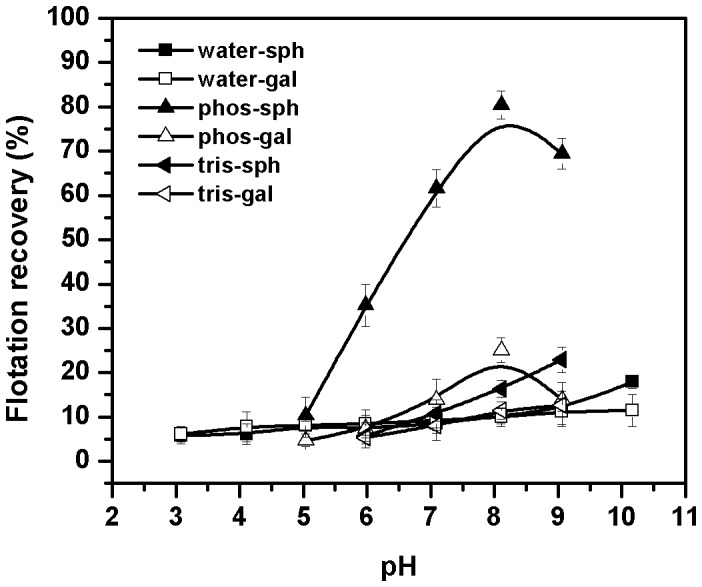
Effect of buffering of cells on the flotation recovery of sphalerite and galena.

*B.circulans* suspended in water was tested for its capacity to float sphalerite or galena as a function of pH of the bacterial cell suspension. The lack of significant floatability of either sphalerite or galena throughout the pH range tested, prompted the exploration of the circumstances under which the bacteria could float the minerals (Fig.1). Since most biological reactions operate under buffered conditions [9], the role of anionic and cationic buffer systems was evaluated in the flotation process. Curiously, only cells suspended in anionic buffers promoted the flotation of sphalerite while cationic buffers did not aid the flotation process. The maximum flotation of sphalerite was observed with phosphate buffer (pH 8.0). Fig. 1 shows a representative trend obtained with an anionic buffer (phosphate) and a cationic buffer (tris-HCl). The intriguing and antagonistic role of the anionic and cationic buffers is presumably related to the nature of the bioreagent (see Discussion). Based on these observations, all the subsequent flotation studies were carried out in the presence of bacterial cells suspended in phosphate buffer (pH 8.0). Buffers per se failed to enhance the flotation recovery of minerals beyond background levels (5%). In the case of galena, neither anionic nor cationic buffers could aid its flotation. These observations indicate that the presence of a component on the surface of *B.circulans* cells which aids the flotation of sphalerite but not galena.

### Nature of the Bioreagent Aiding Sphalerite Flotation

The cell wall architecture of Gram positive bacteria such as *Bacillus* is made up of membrane proteins and a thick peptidoglycan layer connected by wall teichoic acids [10]. Encouraged by the high flotation levels of sphalerite but not galena in the above experiments, an enzymatic method was adopted to identify the nature of the bioreagent(s) involved in the above process.

**Figure 2:**
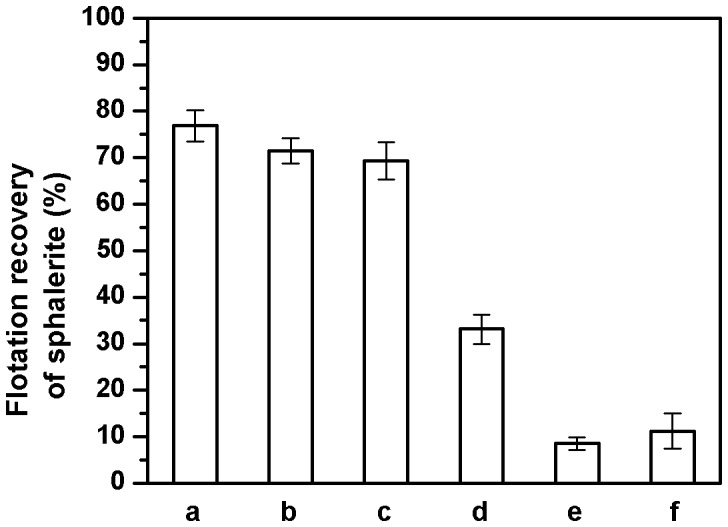
Flotation recovery of sphalerite in the presence of bacterial cells, before and after various enzymatic treatments. a - untreated cells; b - proteinase K treated; c - lysozyme treated; d - phosphate deficient medium; e - purified teichoic acid; f – DNase 1 treated.

To this end, live cells were treated with enzymes to digest away one surface component at a time. The effect of its presence or absence on the flotation of sphalerite was then evaluated. It is pertinent to mention that the enzymatic treatments did not affect the viability of the cells. As shown in Fig. 2, treatment of cells with either proteinase K or lysozyme did not reduce the flotation recovery of sphalerite significantly compared with the untreated control cells. This suggested that the cell surface component aiding flotation is non-proteinaceous and non-polysaccharide in nature. Teichoic acid, which is another major component of cell walls could not be assayed as above, as no enzymatic activity degrading teichoic acid was available commercially. As phosphate is one of the principal constituents of teichoic acids [11] an indirect method of culturing *B.circulans* cells in a phosphate deficient medium was resorted to address the question of its involvement in the flotation process. Cells grown in such a deficient medium showed considerably reduced growth and low teichoic acid content, which are in agreement with similar observations reported earlier [12]. The flotation recovery of sphalerite by these cells was reduced to about 30%, but not inhibited completely (Fig.2). This result suggested that, plausibly, some phosphate-dependent component was aiding flotation. If teichoic acid indeed were to be aiding flotation, purified teichoic acid would be expected to show high flotation recovery of sphalerite, similar to normal cells. But when purified teichoic acid was used in the flotation assay, the recovery of sphalerite was very low (∼10%). This confirms that teichoic acids were not involved in the flotation of sphalerite.

A major phosphate-dependent constituent of cells is nucleic acids. Several bacterial species are known to secrete nucleic acids on to their cell surface. This double stranded DNA has been designated as extracellular DNA (eDNA) to distinguish it from genomic DNA, which is intracellular in location [13]. Surprisingly, this aspect of bacterial cell surface has not received sufficient attention. In contrast to the above enzymatic treatments, it is noteworthy that DNase 1 treatment of cells markedly reduced the flotation recovery of sphalerite to 10%. These experiments clearly indicate that the bioreagent responsible for sphalerite flotation is a non-teichoic acid entity, which is proteinase K and lysozyme resistant, but DNase 1 sensitive.

### Selective Flotation of Sphalerite from a Sphalerite – Galena Mineral Mixture by Thermolysed Cells and its Fractionated Components

**Figure 3:**
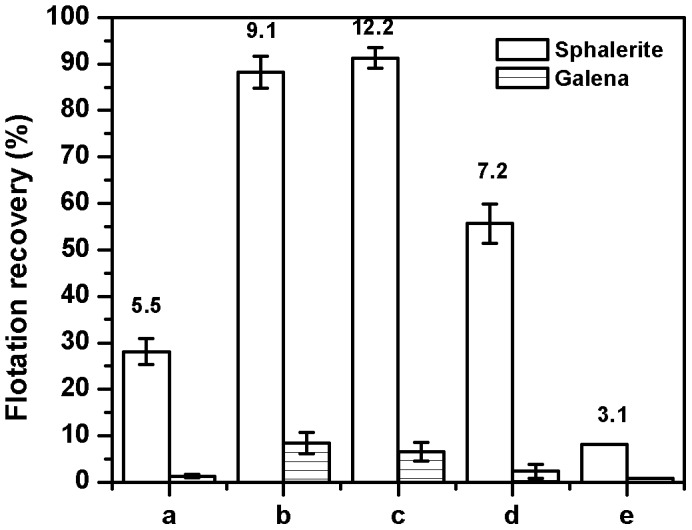
Selective flotation of sphalerite and galena in the presence of unfractionated or thermolysed and fractionated components of *B.circulans.* a - normal cells; b - thermolysed cells; c - thermolysed cell-free supernatant; d - thermolysed cell pellet; e – DNase 1 treated thermolysed cell-free supernatant. The numbers above the bar chart indicates the selectivity index (S.I) values.

In the above context, for any reagent to be considered promising for further development, it should be able to selectively float the mineral of interest from a mineral mixture. For this study, a 1∶1 mixture of sphalerite and galena was chosen and the selectivity index was calculated based on equation (1). As shown in Fig.3 normal cells are able to partially float sphalerite from the mineral mixture with a selectivity index of 5.5. In this case, sphalerite and galena were floated to the extent of 28% and of 3% respectively. This recovery is much lower than that observed with the flotation of sphalerite by cells, where almost 80% of the mineral was floated (Fig.1). The difference between the two observations could be due to the high level of unproductive adsorption of cells on to galena vis-à-vis sphalerite [14]. In order to enhance the selective recovery of sphalerite it was evaluated whether viability or intactness of *B.circulans* cells is necessary to float sphalerite. If the observations of the previous section were to be true, thermolysed cells should show higher selective flotation recovery of sphalerite from the mineral mixture compared with live cells. Thermolysis results in rupturing of the bacterial cell structure and releasing all the intracellular contents into the medium [15]. One of the chief components of interest, vis-à-vis sphalerite flotation, which is released into the medium is genomic DNA. Thus, higher flotation recovery of sphalerite is expected to be due to the combined effect of the intracellular and the extracellular DNA that results from cell disruption.

As shown in the Fig. 3 this expectation was borne true. Interaction with the thermolysed cells resulted in the flotation recovery of 90% for sphalerite and 10% for galena to give a selectivity index of 9.1. Thermolysed cells were subsequently fractionated and assayed for the selective flotation of sphalerite. This was carried out to determine the extent of partitioning of the bioreagent into the supernatant and pellet fractions. The thermolysed cell-free supernatant behaved slightly better than the thermolysed cells in that 92% of sphalerite and 7% of galena were floated to give a selectivity index of 12.2. The cell pellet fraction, however, yielded a flotation recovery of 55% of sphalerite and 5% of galena with a lower selectivity index of 7.2. Furthermore, DNase 1 treatment of the thermolysed cell-free supernatant significantly reduces the selective flotation of sphalerite, again indicating that DNA aids sphalerite flotation. This experiment clearly demonstrates that *B.circulans* cells or its constituents selectively float sphalerite from a sphalerite-galena mixture. Disruption of cell structure by thermolysis enormously enhances the flotation recovery of sphalerite from the mineral mixture and a majority of the entity aiding sphalerite flotation is released into the supernatant.

### Purified DNA Floats Sphalerite but not Galena

**Figure 4:**
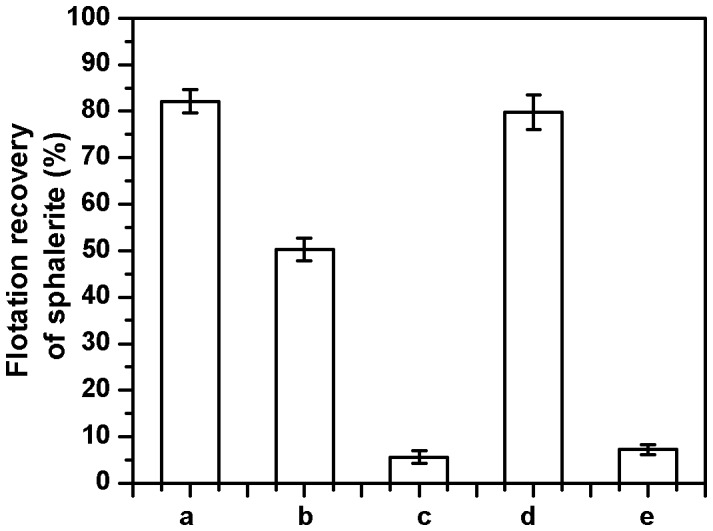
Effect of the strandedness of DNA on the flotation of sphalerite. a - thermolysed cell-free supernatant; b - genomic DNA (2 mg of dsDNA); c – DNase 1 treated genomic DNA; d - ssDNA (2 mg); e - DNase 1 treated ssDNA.

In order to conclusively prove that DNA is the entity aiding sphalerite flotation, genomic DNA purified from *B.circulans* cells was used for flotation tests. As shown in Fig. 4 using purified genomic DNA as collector (2 mg dsDNA which is the equivalent of that present in thermolysed cell-free supernatant), the flotation recovery of about 50% of sphalerite could be achieved. Treatment of genomic DNA with DNase 1 prior to interaction with sphalerite, significantly diminishes its flotation (<10%). However, using the same amount of thermolysed genomic DNA (ssDNA), greater than 80% recovery of sphalerite could be achieved. This is comparable to that obtained with thermolysed cell-free supernatant. The conversion of dsDNA to ssDNA leads to a effective doubling of the bio-collector concentration. As observed previously, treatment of the thermolysed genomic DNA with DNase 1 prior to interaction with sphalerite, almost completely retards its flotation (<10%). This experiment clearly establishes that the reagent involved in the flotation process in thermolysed cells or its supernatant is ssDNA and the lower flotation recovery obtained with viable cells is due to the dsDNA present on its cell surface. Studies carried out by others regarding the source of this eDNA on bacterial surfaces have indicated that it arises from the intercellular genomic DNA via multiple ways [16]. This experiment also establishes that ssDNA acts as high capacity bio-collector compared to equivalent amounts of dsDNA in the flotation of sphalerite.

**Figure 5:**
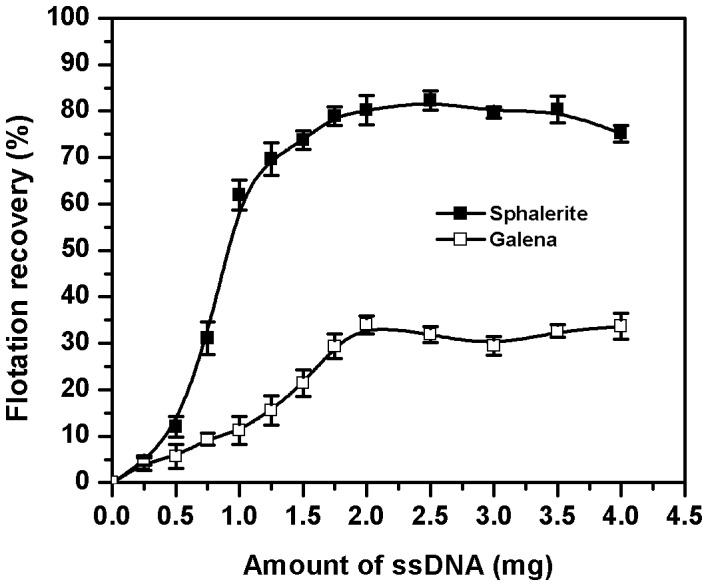
Flotation recovery of sphalerite and galena as a function of the ssDNA concentration.

Fig. 5 shows the concentration dependent recovery of sphalerite and galena by purified ssDNA in a flotation experiment. The flotation recovery of sphalerite reaches a maximum (85%) in the presence of 2 mg of ssDNA, beyond which it remains unchanged. The flotation of galena also shows a steady marginal increase up to about 2 mg of ssDNA and then attains a plateau. However, the maximum flotation of galena observed is 30%. These profiles reflect largely the relative recoveries of sphalerite and galena observed with intact cells or its components.

### Amphipathic Nature of ssDNA Facilitates Sphalerite Flotation

**Figure 6:**
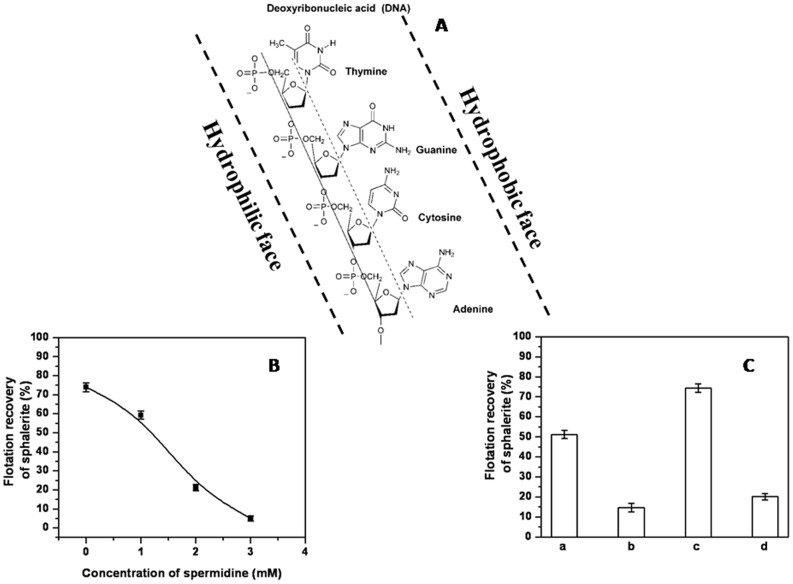
Schematic diagram of the amphipathic nature of ssDNA. (A) Amphipathic structure of ssDNA indicating the hydrophobic and the hydrophilic faces; (B) - Effect of spermidine concentration on the ssDNA mediated flotation of sphalerite; (C) - Effect of depurination of DNA on the flotation of sphalerite a - dsDNA; b - depurinated dsDNA; c - ssDNA; d - depurinated ssDNA.

It thus becomes of interest to understand the mechanism of ssDNA induced flotation of sphalerite. An examination of the structure of DNA indicates its amphipathic nature with the polyphosphate groups aligned on one face of the long axis and the hydrophobic bases aligned on the opposite face (Fig. 6a). At pH 8.0, DNA behaves as a polyanionic species [17]. In the case of dsDNA, the negatively charged phosphates are present on the outside of the double stranded helix while the stacked hydrophobic bases are buried on the inside of the helix due to base pairing of the two strands. Thus pairing of strands evidently reduces its amphipathic nature. In contrast to this, the unpaired state of ssDNA makes its amphipathic nature more conspicuous. The higher intrinsic amphipathic nature, along with an increase in the effective bio-collector concentration may be responsible for the higher flotation recovery of sphalerite by ssDNA vis-à-vis dsDNA (Fig. 4). We hypothesized that the above structure could lead to strong interaction between sphalerite and ssDNA such that the phosphate groups could interact with the sphalerite while the aromatic bases could align across the axis to form the hydrophobic surface. Thus ssDNA could act as a polymeric heteropolar collector. To validate this hypothesis we evaluated the involvement of both the surfaces of this amphipathic molecule in the flotation process.

The hydrophilic face containing the phosphate groups is not amenable to removal, as it constitutes the backbone of the DNA polymer. However, the effect of the hydrophilic face of the DNA molecule can be reduced by the electrostatic interactions of phosphate groups with inorganic and other small cationic molecules in a concentration dependent manner [18], [19]. The neutralization of the polyanionic character of ssDNA with spermidine (a cellular cationic small molecule) to abolish or interfere in the flotation recovery of sphalerite was evaluated. Essentially, there is a competition between the mineral and spermidine for the polyanionic ssDNA. It was ensured that the concentration of spermidine used in this experiment (upto 3 mM) is several fold lower than that known to precipitate DNA (>10 mM) [20]. As shown in Fig 6b, with increasing concentration of spermidine the flotation of sphalerite continuously decreases. This attests to the need for the charged phosphate groups in ssDNA for effective flotation to take place.

While the polyanionic ssDNA interacts with the mineral, through chemical and electrostatic forces, the aromatic bases present on the outer surface presumably provide the necessary hydrophobicity for the flotation process. To ascertain if this indeed were to be the case, a considerable fraction of the bases was removed by depurination before interacting with the mineral. An otherwise continuous stretch of hydrophobic face of DNA appears discontinuous and punctured with patches of hydrophilic holes following acid depurination. If hydrophobicity of DNA is indeed necessary for the flotation of sphalerite, any reduction in this property would negatively impact the flotation process. The effect of depurination of dsDNA and ssDNA on the flotation of sphalerite was assessed. Previous estimations had indicated that close to 50% of the bases are converted to apurinic acid by this treatment [21]. As anticipated, the flotation recoveries were drastically reduced after depurination of either dsDNA or ssDNA (Fig.6c). This clearly indicated that the aromatic entities of ssDNA imparted the necessary hydrophobicity for the flotation process. The foregoing data demonstrates that the amphipathic nature of ssDNA is absolutely essential for the successful flotation of sphalerite.

### Non-DNA Components of Lysed Cells Act as a Depressant for Galena in the Selective Flotation Process

**Figure 7:**
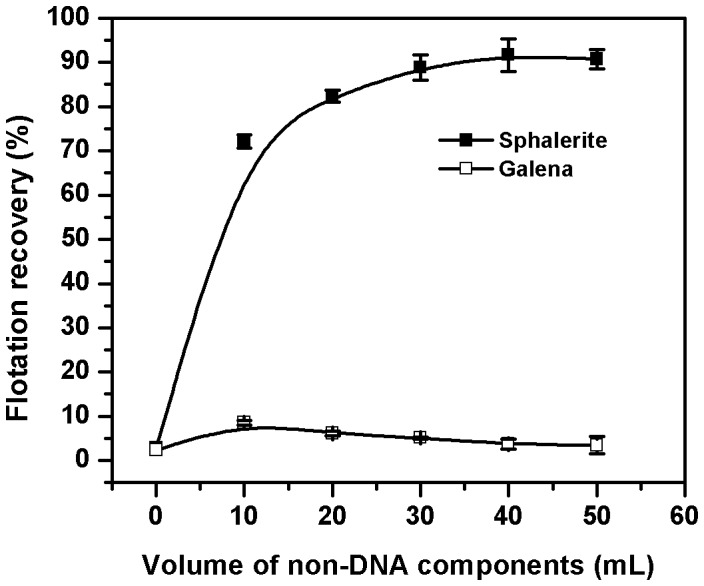
Flotation recovery of sphalerite and galena during reconstitution of the selective flotation of sphalerite with a fixed amount (2 **mg) of ssDNA and varying amounts of DNase 1 treated non-DNA components of cells.**

Having identified DNA, or more specifically ssDNA, as the bio-collector responsible for the high flotation recoveries of sphalerite, it becomes of interest to reconstitute the process of selective flotation. It has been observed that ssDNA could float sphalerite but not galena. Surprisingly, in contrast to the thermolysed cell-free supernatant, ssDNA per se showed no selectivity towards sphalerite in the sphalerite-galena mixture. In fact, hardly any flotation of either mineral was observed (Fig.7). This indicated that other (non-DNA) components of the thermolysed supernatant may also be involved in the selective flotation of sphalerite. However, by adding varying amounts of DNase 1 treated thermolysed cell-free supernatant to a defined amount of ssDNA prior to interaction with the mineral, the degree of selective flotation of sphalerite from the sphalerite-galena mixture could be restored to that observed in the case of sphalerite when present alone. Importantly, DNase 1 treated thermolysed cell-free supernatant by itself did not selectively float sphalerite (refer Fig.3). This experiment clearly reveals that ssDNA which can float sphalerite is ineffective for the selective flotation of sphalerite from the sphalerite-galena mixture. It needs other non-DNA components of the thermolysed cell-free supernatant, bulk of which appears to be polyanionic, to selectively float sphalerite from its mixture with galena. These non-DNA polyanions presumably compete with ssDNA for adsorption on to the mineral surface and prevent the non-specific and non-productive adsorption of ssDNA on galena. These experiments clearly demonstrate the separation of bio-collector and bio-depressant functions between ssDNA and the non-DNA components of thermolysed cells. Reconstitution data presented in Table - 1 clearly shows that the selectivity index values with other *Bacillus* species used in the study follow a similar trend during the selective flotation of sphalerite from the mineral mixture.

**Table 1:** Organism dependent Selectivity Index values during reconstitution of selective flotation tests using fixed amount (2 mg) of ssDNA.

Volume of non-DNA components (mL)	*B.circulans*	*B.megaterium*	*B.subtilis*	*P.polymyxa*
0	1.1	1.7	1.3	1.4
10	5.3	3.1	2.7	3.6
20	8.5	5.6	2.9	5.9
30	12.2	8.3	5.1	8.6
40	16.5	15.3	9.4	10.8
50	16.9	19.1	9.9	12.0

## Discussion

Traditionally, DNA has always been ascribed biological roles which are central to all life forms. Of late, some non-biological roles such as its use in nano-technology has been recognized [22], [23]. In this communication, we have identified that DNA, or more specifically ssDNA, acts as bio-collector in the selective floatation of sphalerite from a sphalerite-galena mineral mixture. This indeed is a novel property of DNA and one that is very different from the biological properties of DNA identified so for. We have demonstrated that the amphipathic nature of ssDNA facilitates the flotation process.

Earlier studies from this laboratory have shown that the capacity of galena to adsorb *P. polymyxa* cells is an order of magnitude higher than that of sphalerite [14]. This situation likely appears to be true for *B.circulans* also. When pure ssDNA is used in the selective flotation of sphalerite from a sphalerite-galena mixture, the flotation recovery is found to be highly reduced compared to that with sphalerite alone. Addition of several fold excess of ssDNA does not significantly enhance the flotation recoveries of sphalerite from the mixture, presumably due to the higher preferential adsorption of ssDNA by galena over sphalerite. However, in the presence of a large excess of other polyanionic species in the thermolysed cell-free supernatant (which comprises of all the cellular non-DNA components), ssDNA is largely left free to bind with sphalerite and aid its flotation, while the non-DNA components preferentially binds with galena. In this context, polysaccharides have been shown to act as a depressant in the flotation of sulfide minerals [24], [25]. Reconstitution studies presented above indicate that the ratio of the bio-collector and bio-depressant probably determines the flotation recoveries of sphalerite from the mineral mixture.

Based on the above investigations, we present a model for the flotation of sphalerite from a sphalerite-galena mixture. This process needs three essential components.

1. an anionic buffer component
2. a bio-collector (ssDNA) which facilitates the flotation process
3. bio-depressants which retard the flotation process. This comprises of teichoic acids and polysaccharides, which are essentially non-amphipathic polyanions.

The antagonistic role of anionic and cationic buffers, rather than its pH, in the flotation of sphalerite calls for an explanation. A probable reason for the negative effect of cationic buffer on the flotation process may be due to the competition of the mineral surface with metal cations for interaction with the poly-anionic ssDNA. The buffer cations being far in excess, probably out compete the metals to bind ssDNA. In contrast to this, an anionic buffer such as phosphate compete with the poly-anionic ssDNA for the metal surface. The poly-anionic nature of ssDNA compared to the monomeric nature of the buffer anion leads to a synergistic interaction with the metal cations on the mineral surface. Thus, the presence of relevant factors that lead to better mineral-ssDNA interaction and the resultant induction of hydrophobicity on the mineral surface has consequences for high flotation recovery.

Metal cations have been shown to have two modes of interaction with the constituents of DNA, viz., phosphates and bases. Pb is known to interact more avidly with both the phosphate backbone and aromatic bases than Zn [26]. Additionally, the lattice structure of sphalerite rather than galena may be more amenable to a favorable interaction with ssDNA. For these reasons, ssDNA may not be able to induce sufficient hydrophobicity to galena, leading to a low recovery.

In summary, a novel bio-collector property of ssDNA for sphalerite flotation has been demonstrated. Furthermore, the twin presence of the bio-collector and bio-depressants is absolutely essential to achieve higher selective flotation of sphalerite from a sphalerite-galena mixture.

## Materials and Methods

Mineral samples of sphalerite and galena were obtained from Wards Natural Science Establishment (USA) and Alminrock Indscer Fabriks (India) respectively. Mineralogical studies as well as X-ray powder diffraction data indicated that the samples were of high purity (99.8%). The above samples were dry ground using a porcelain ball mill and then sieved through BSS sieves.

*B. circulans* (NCIM 2160), *B. subtilis* (NCIM 2063), *B. megaterium* (NCIM 2087), *P. polymyxa* (NCIM 2539) and *E. coli* K12 (NCIM 2674) used in this study were obtained from the National Collection of Industrial Microorganisms (NCIM), National Chemical Laboratory, Pune. The bacteria were cultured using the Bromfield medium as described elsewhere [27]. Phosphate deficient Bromfield medium had a phosphate content of 0.05 g/L. Spermidine was obtained from Sigma Aldrich while the enzymes used in this study, viz., proteinase K, lysozyme and DNase 1 were obtained from Bangalore Genei.

### Harvesting, Cell Fractionation and Extraction of Genomic DNA

Cells were harvested from fully grown bacterial cultures (48 h) by centrifugation at 5000 rpm for 20 min at 4°C. Bacterial cells (1×10^10^) washed and suspended in 0.1 M phosphate buffer (pH 8.0) were used directly or thermolysed in a water bath at 100°C for 30 min and cooled before being used in the flotation tests. The thermolysed cell suspension was centrifuged at 10,000 rpm for 20 min at 4°C to obtain the soluble thermolysed cell-free supernatant and insoluble thermolysed cell pellet. The thermolysed cell-free supernatant was assayed directly while the insoluble pellet was suspended in 0.1 M phosphate buffer before assaying.

Extraction of genomic DNA was essentially carried out as per the procedure described elsewhere [28]. Tris HCl (pH 8.0) was however replaced by 0.1 M phosphate buffer (pH 8.0). In this communication, genomic DNA, which is double stranded, has been referred to interchangeably as dsDNA and thermolysed genomic DNA, which is single stranded, as ssDNA.

### Enzymatic Treatment of Cells

Bacterial cells (1×10^10^) suspended in 0.1 M phosphate buffer (pH8.0) were treated with enzymes at the indicated concentrations (1 mg/mL proteinase K, 10 mg/mL lysozyme or 200 units DNase 1 in 10 mM MgCl_2_) separately for 4 h. After treatment, the cells were centrifuged at 5000 rpm for 20 min, washed and suspended in 0.1 M phosphate buffer before being used in the flotation experiments.

### Extraction of Teichoic Acid from Cells

This was carried out as described elsewhere [29]. Purified teichoic acid was dissolved in 0.1 M phosphate buffer before using it in the flotation tests of sphalerite or galena.

### Flotation of Sphalerite or Galena and Selective Flotation of Sphalerite from a Mixture of Sphalerite and Galena

1 g of sphalerite or galena of size (−150+100) µm was used for the flotation experiments. For selective flotation a mixture of sphalerite and galena of size (−150+100) µm in the ratio of 1∶1 (0.5 g each) was used. Pure mineral or the mineral mixture was conditioned with the chosen reagent (whole cells/thermolysed cells/thermolysed cell-free supernatant/thermolysed cell pellet) in 0.1 M phosphate buffer at pH 8.0 for 30 minutes prior to the flotation process. After interaction the suspension was transferred to a modified Hallimond tube [30]. Nitrogen gas at a flow rate of 40 mL/min was passed through the cell and the flotation was carried out for 3 minutes. The concentrate and tailing fractions were separately filtered, dried and weighed. Lead and zinc contents in the concentrate and tailing fractions were estimated using an Atomic Absorption Spectrometer (AAS, Thermo Electron Corporation MM series). Selectivity Index was calculated according to Gaudin’s formula [31] as shown in equation (1).
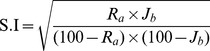
(1)where,R_a_ – Percentage recovery of sphalerite in the float fraction,J_b_ – Percentage recovery of galena in the tailings fraction.

### Depurination of DNA and Interaction of ssDNA with Spermidine

Depurination of DNA was carried out as described [21]. Genomic DNA (2 mg) was adjusted to pH 1.6 with HCl in the presence of 50 mM NaCl and 1.5 mM of sodium citrate and dialyzed for 15 hours at 37°C. The dialysate was either thermolysed or used directly for the flotation of sphalerite.

ssDNA obtained from thermolysis of 2 mg of dsDNA in 0.1 M phosphate buffer (pH 8.0) was interacted with varying concentrations of spermidine for 2 h at 37°C as described [32] prior to its use in the flotation of sphalerite.

### Reconstitution of the Selective Flotation Process

Selective flotation was reconstituted by adding ssDNA (obtained from 2 mg of thermolysed genomic DNA) or ssDNA together with varying amounts of DNase 1 treated thermolysed cell-free supernatant to the mineral mixture. The flotation procedure as described earlier was adopted.

All the above experiments were carried out atleast three times to determine the standard error.
